# Endoscopic reconstruction of complete post-radiation esophageal and neopharyngeal obliteration

**DOI:** 10.1055/a-2839-9584

**Published:** 2026-04-15

**Authors:** Georgios Mavrogenis, Konstantinos Markoglou, Apostolos Mantides, Nikolaos Samarentsis, Georgios Theoharis, Paraskevas Gkolfakis, Konstantinos Triantafyllou

**Affiliations:** 1 Third Space Endoscopy Unit, Mediterraneo Hospital, Athens, Greece; 2 Hepatogastroenterology Unit, Athens Naval Hospital, Athens, Greece; 3 Hepatogastroenterology Unit, Second Department of Propaedeutic, Internal Medicine, Medical School, National and Kapodistrian University of Athens, Attikon University General Hospital, Athens, Greece

Complete post-radiation esophageal obliteration is a rare complication traditionally managed surgically. Hereby, we present a step-by-step endoscopic approach using submucosal tunneling and sequential dilation, while protecting the gastrostomy orifice through endoscopic gastropexy 1 2 .

**Fig. 1:**
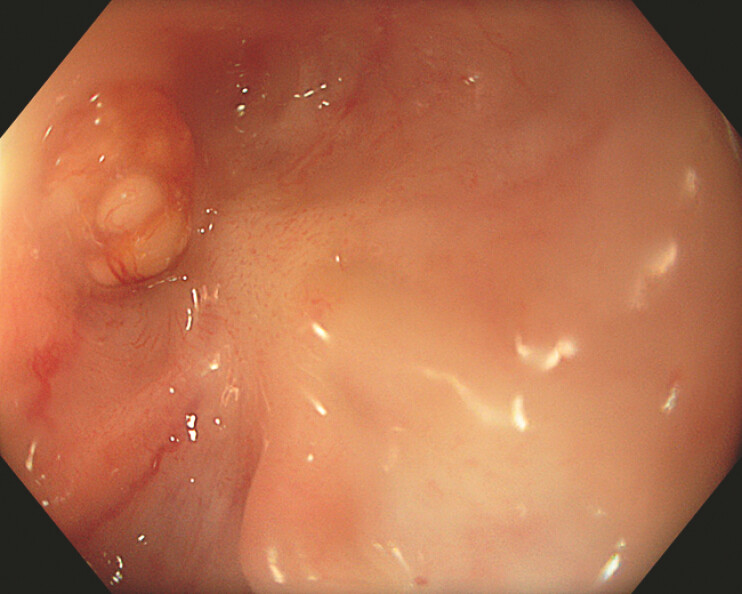
A retrograde view of the obstruction.

A 75-year-old man presented with complete dysphagia due to a 6-cm obliteration of the cervical esophagus and neopharynx ( Fig. 1 ) following laryngectomy and chemoradiation for laryngeal cancer. Nutritional support was maintained via a gastrostomy ( Video 1 ).

Video demonstration of the reconstruction of the esophagus and neopharynx.Video 1

**Fig. 2:**
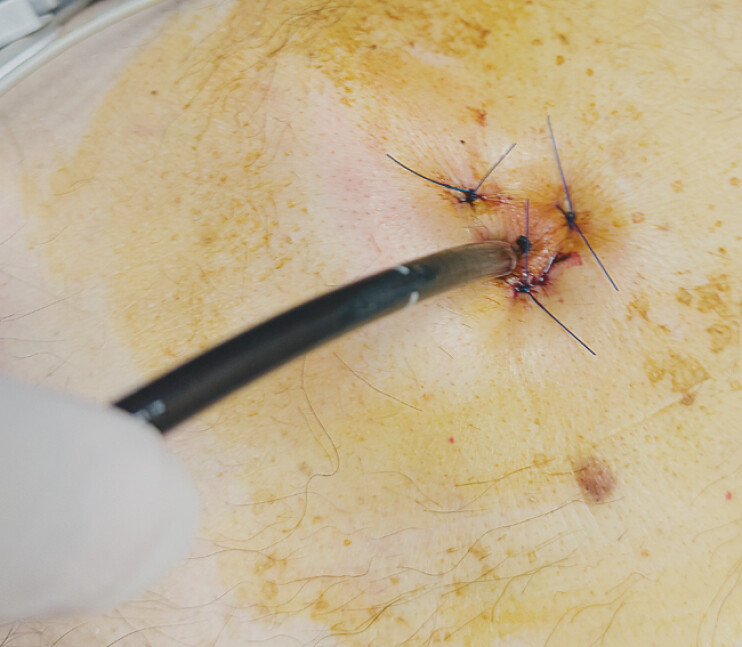
Gastropexy secured the gastrostomy fistulus tract.

**Fig. 3:**
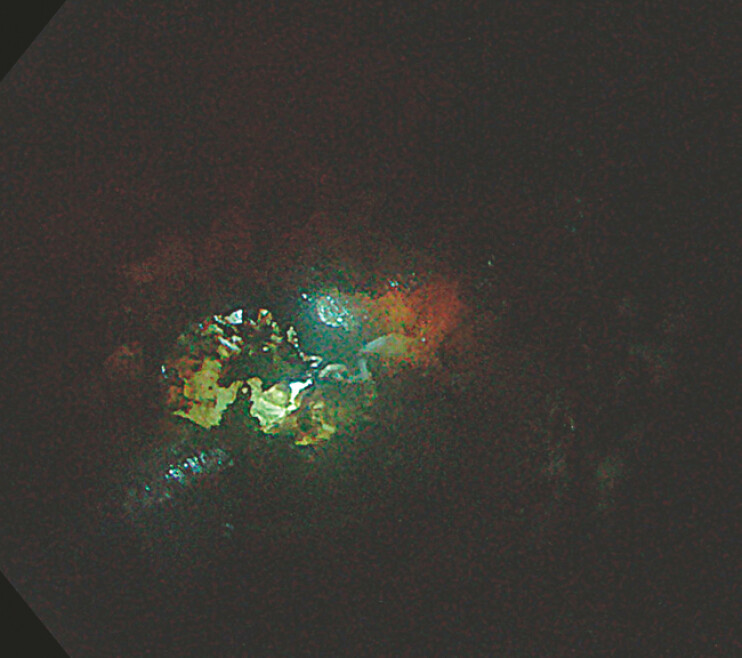
Transillumination via an orally advanced endoscopy guided the path towards the neopharynx.

**Fig. 4:**
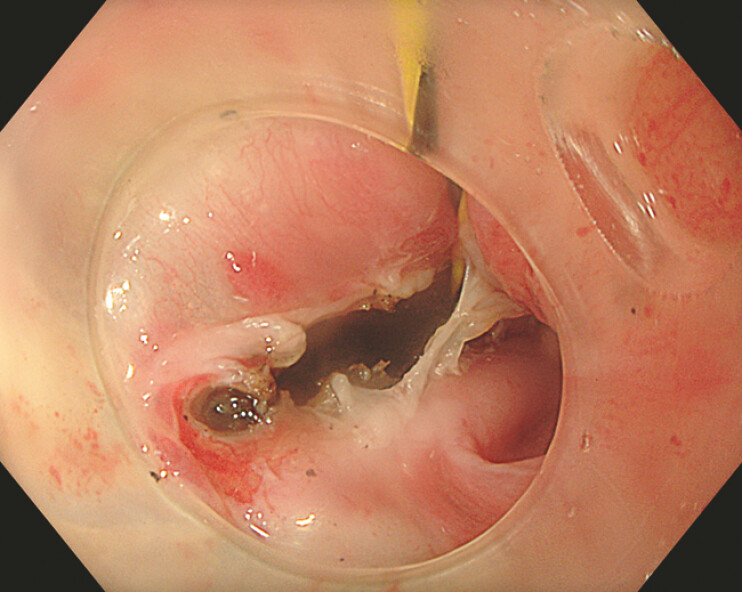
An antegrade view of the rendezvous.

**Fig. 5:**
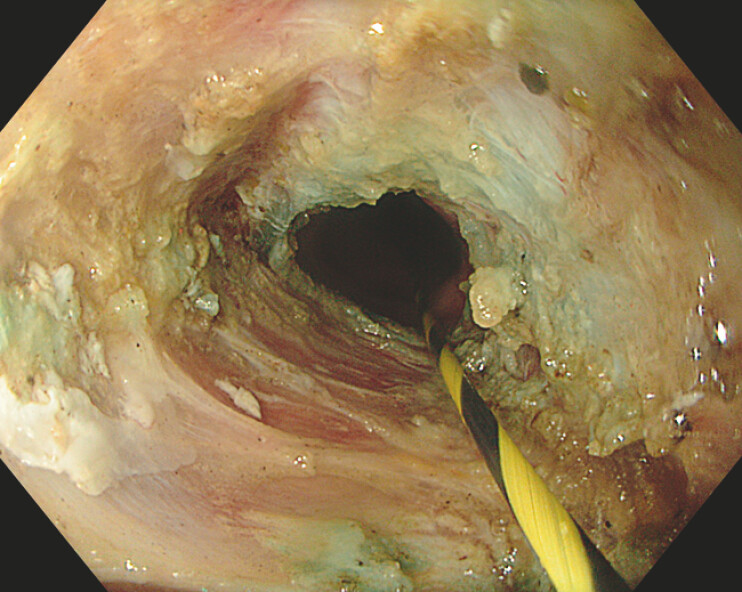
An antegrade view of the reconstructed lumen.

After a multidisciplinary discussion, endoscopic reconstruction was planned. Initially, the gastrostomy tract was secured with endoscopic gastropexy ( Fig. 2 ). Four fixation sutures were placed to minimize the risk of perforation. Three weeks later, the gastrostomy site was dilated to 18 mm, allowing retrograde insertion of a standard gastroscope. Stepwise dissection of the fibrotic tissue was performed using a ball-type knife in Endocut mode, with spray coagulation applied to loose tissue. A retrograde tunnel was progressively created toward the neopharynx by following the axis of the muscle layer. Upon reaching the proximal esophagus, transillumination from a perorally advanced gastroscope enabled an endoscopic rendezvous ( Fig. 3 ). A guidewire was placed to delineate the tract and facilitate antegrade dissection ( Fig. 4 ). Final balloon dilation to 15 mm allowed the passage of a standard endoscope ( Fig. 5 ).

The patient was discharged after 48 hours, uneventfully apart from transient cervical emphysema. The nasogastric tube was maintained for 2 weeks, while the gastropexy sutures were removed 1 month thereafter. No steroid injection was administered due to its limited efficacy. Furthermore, no stent was placed because of concerns regarding pain, local discomfort, and the risk of fistula formation. The PEG tube was maintained in situ for safety reasons. During 9 months of follow up, he continues monthly serial balloon dilations up to 15 mm and tolerates soft solids.

Endoscopy_UCTN_Code_TTT_1AO_2AG Endoscopy_UCTN_Code_TTT_1AO_2AG_3AZ
